# Cerebral blood flow velocity and oxygenation in neonatal aortic arch repair at two perfusion temperatures

**DOI:** 10.1093/ejcts/ezad220

**Published:** 2023-06-06

**Authors:** Lucy E M Finnigan, Robyn Lotto, Helen Jones, Attilio Lotto

**Affiliations:** Division of Cardiovascular Medicine, Radcliffe Department of Medicine, University of Oxford, Oxford, UK; Allied Health and Nursing, Liverpool John Moores University, Liverpool, UK; Research Institute for Sport and Exercise Sciences, Liverpool John Moores University, Liverpool, UK; Heart Centre, Alder Hey Children’s NHS Foundation, Liverpool, UK

**Keywords:** Transcranial doppler ultrasound, Near-infrared spectroscopy, Temperature changes, Aortic arch repair, Neurological deficit

## Abstract

**OBJECTIVES:**

(i) To monitor cerebral blood flow velocity (CBFv) throughout aortic arch repair surgery and during the recovery period. (ii) To examine the relationship between transcranial doppler ultrasound (TCD) and near-infrared spectroscopy (NIRS) during cardiac surgery. (iii) To examine CBFv in patients cooled to 20°C and 25°C.

**METHODS:**

During aortic arch repair and after surgery, measurements of TCD, NIRS, blood pH, pO_2_, pCO_2_, HCO_3_, lactate, Hb, haematocrit (%) and temperature (core and rectal) were recorded in 24 neonates. General linear mixed models were used to examine differences over time and between two cooling temperatures. Repeated measures correlations were used to determine the relationship between TCD and NIRS.

**RESULTS:**

CBFv changed during arch repair (main effect of time: *P* = 0.001). During cooling, CBFv increased by 10.0 cm/s (5.97, 17.7) compared to normothermia (*P* = 0.019). Once recovering in paediatric intensive care unit (PICU), CBFv had increased from the preoperative measurement by 6.2 cm/s (0.21, 13.4; *P* = 0.045). CBFv changes were similar between patients cooled to 20°C and 25°C (main effect of temperature: *P* = 0.22). Repeated measures correlations (rmcorr) identified a statistically significant but weak positive correlation between CBFv and NIRS (*r* = 0.25, *P*≤0.001).

**CONCLUSIONS:**

Our data suggested that CBFv changed throughout aortic arch repair and was higher during the cooling period. A weak relationship was found between NIRS and TCD. Overall, these findings could provide clinicians with information on how to optimise long-term cerebrovascular health.

**Figure:**
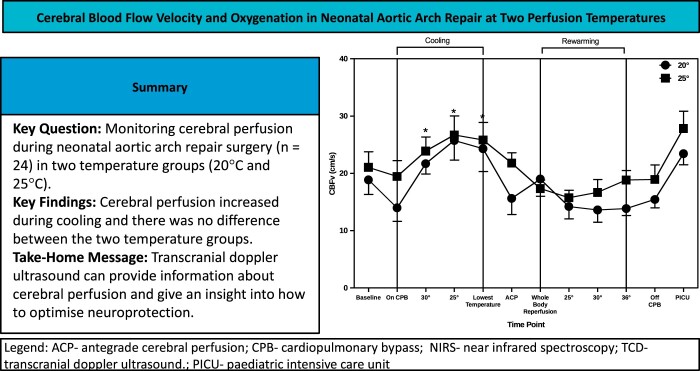

## INTRODUCTION

Survival rates in patients born with congenital heart disease have increased due to improvements in clinical care and treatment options [1]. However, a significant percentage of survivors exhibit neurological deficit [2]. The highest rates of deficit are found in those with severe left-sided lesions such as hypoplastic or interrupted aortic arch [3]. This deficit is likely multifactorial and a potential contributing factor is disruptions to cerebral blood flow (CBF) during surgery [4]. There are many factors during aortic arch repair that can lead to instances of both cerebral tissue hypoxia and regional hypoperfusion. This includes the establishment of anaesthesia, the cessation of cardiac function, the use of cardiopulmonary bypass (CPB) and the induction of deep hypothermic circulatory arrest (DHCA) [5]. Making the monitoring of cerebral perfusion during aortic arch repair imperative in identifying time periods of disrupted flow, in order to optimise neurological protection strategies.

Currently, neurological monitoring practises vary considerably across centres with no universal guidelines [6]. Several strategies are implemented for neurological protection which aim to reduce neural activity in the brain, this includes whole body cooling via CPB perfusion. However, there is a lack of empirical evidence for the optimum minimum temperatures used during whole body cooling. The target temperature is often based on surgical complexity and surgeon preference with temperatures ranging from 16°C to 28°C. Another strategy to ensure adequate perfusion is the constant monitoring of near-infrared spectroscopy (NIRS). NIRS is a simple, cost-effective measurement of cerebral oxygenation. However, the shortcomings of NIRS have been previously documented, including high levels of inter-device variability, issues related to absolute versus relative saturations and variability of oxygen saturation targets/thresholds in clinical settings [7].

More in-depth measurements of cerebral perfusion are available such as transcranial doppler (TCD) ultrasound, which provides CBF velocity (CBFv) of the major cerebral vessels. Previously, both TCD and NIRS were employed during neonatal aortic arch repair during full flow CPB. Periods of hyperperfusion were reported which NIRS was unable to detect [8]; however, measurements were not acquired throughout the whole procedure. Whereas a case study measured TCD and NIRS in one male infant (10 days old) throughout the entire procedure. An important observation was values in TCD and cerebral oxygenation dropped to near zero at two different time points during DHCA [9], suggesting the patient was subjected to possible periods of cerebral ischaemia. This suggests that further research is warranted in a larger population to identify periods of hypo- and hyperperfusion. Therefore, the aim of this study was to continuously monitor CBFv throughout aortic arch repair surgery and during recovery in paediatric intensive care unit (PICU). The secondary aim was to examine the relationship between NIRS and TCD at each time point during cardiac surgery. The final aim was to examine difference in CBFv between patients cooled to 20°C and 25°C as part of the neuroprotection regime.

## PATIENTS AND METHODS

### Ethics statement

Formal written informed consent was obtained from the parent/guardian by a member of the surgical team prior to the procedure. Ethical approval was obtained from the North West Liverpool East Research Ethics Committee (Approval number 17/NW/0249). The study was registered with ClinicalTrials.gov (Identifier: NCT03047876). Neonates of infants (<1 year old) who required surgery for aortic arch repair, interrupted aortic arch repair or the Norwood procedure were included. Exclusion criteria consisted of patients in critical clinical conditions on inotropic support and in metabolic acidosis. Moreover, parents unable to give consent were not considered for the study. Other exclusion criteria were patients with previous documented neurological damage and those suffering from conditions which have been known to affect CBFv such as significant elevated bilirubin levels (patients requiring phototherapy treatment), sickle cell anaemia and moyamoya disease.

### Patients

Neonates were recruited in a clinical setting from 22 November 2018 until 26 February 2020 at Alder Hey Children’s NHS Foundation Trust. Study size was limited to a single centre where all patients that met the inclusion criteria during the recruitment period were approached to reduce bias. Patients required surgery for hypoplastic aortic arch (*n* = 16), interrupted aortic arch (*n* = 6) and hypoplastic left heart syndrome (HLHS) (*n* = 2). Five patients in total underwent total circulatory arrest but were included as CBFv was comparable to those that did not require circulatory arrest. Patients were split into two groups dependant on lowest temperature achieved during cooling which was 20°C (*n* = 8) or 25°C (*n* = 16; Table 1). Target cooling temperature was predetermined by operating surgeons preference, complexity of the procedure and predicted CPB times.

**Table 1:** Patient characteristics of 2 temperature groups

	20°C	25°C
Mean age (days)	20 ± 16	18 ± 19
Height (cm)	48.7 ± 4.8	52.3 ± 3.9
Weight (kg)	3.3 ± 0.7	3.6 ± 0.7
Sex (females:males)	1:1	5: 11
Mean cardiopulmonary bypass time (min)	197 ± 45	136 ± 38
Mean cross-clamp time (min)	119 ± 34	88 ± 105
Mean total circulatory arrest time (min)	48 ± 59 (*n* = 2)	5 ± 3 (*n* = 3)
Mean antegrade cerebral perfusion time (min)	78 ± 18	61 ± 23

### Research design

A team of three consultant cardiac surgeons performed the aortic arch repairs with a standardised surgical technique which has previously been described [10]. Measurements were taken using a TCD ultrasound system (DWL, Compumedics, Germany) with a 2 or 4 MHz doppler probe alongside NIRS (ForeSight, Casmed, UK). The results of serial arterial blood gas analysis [pH, pO_2_, PCO_2_, HCO_3_, lactate, Hb, haematocrit (Htc)] were recorded during measurements as well as mean arterial blood pressure, heart rate and CPB flow rates. CBFv were obtained first through the temporal window where the ultrasound probe was directed horizontally. The depth and sample volume were adjusted until the middle cerebral artery (MCA) was found using previously published normative values for the age range [11]. However, during paediatric cardiac surgery access to the temporal window was not always possible without disturbing the surgical field due to the draping and positioning of the patients head. Patients were neonates with an open fontanelle which was accessible throughout the procedure. Therefore, during baseline measurements and before surgery had begun, the temporal window (which was accessible at this time point) was first used as a reference point to ensure the MCA velocity, depth and power was similar from both windows. For the remaining measurements, the probe was placed on the same single vessel (right MCA) through the fontanelle. The same position and the same depth were maintained throughout. Previous research has suggested there is no significant difference between blood flow velocities in left and right middle cerebral vessels or hemispheres during paediatric cardiac surgery if the circle of Willis is intact [12]. All patients had normal cerebral vasculature.

Anaesthetic technique was standard for all patients consisting of intravenous administration of midazolam, fentanyl and inhaled sevoflurane. Two blood pressure monitors were used (femoral and right radial or brachial artery) to assess preductal and postductal arterial pressures. Two NIRS probes were positioned on bilateral frontal area and monitoring started. Once the patient was anaesthetised and prepared for theatre, a ‘baseline’ measurement was recorded. Surgery was performed via median sternotomy, preparation for the procedure included dissection of the head and neck vessels, ascending aorta, aortic arch and descending aorta past the aortic isthmus. Patent ductus arteriosus (PDA) was dissected and encircled with a silk ligature, ready to be ligated upon CPB commencement. CPB was achieved with either a single right atrial cannula or bicaval cannulation in those patients requiring intracardiac repairs. Aortic return was procured in the ascending aorta with a straight perfusion cannula. In patients with a systemic duct dependent circulation such as HLHS or interrupted aortic arch, a second cannula was positioned across the PDA via a purse string on the pulmonary trunk. This provided adequate systemic perfusion when on CPB. In such cases, flow rates were adjusted to provide two-third of the total flow to the lower body, and one-third to the upper body. After heparin administration and with an activated clotting time of more than 300 seconds, CPB was then commenced and cooling towards the target temperature was accomplished. In all cases core temperature was monitored via rectal and nasopharyngeal temperature probes. CPB full flow rates were calculated at 2.6 l/min/m^2^ of body surface area. Body surface area was calculated as the square root of [height (cm)×weight (kg)/3600]. PDA was ligated, and in case of a second cannula, a ligature was snagged around the main pulmonary artery to avoid pulmonary over circulation and relative systemic hypoperfusion.

On initiation of full flow bypass, a CBFv measurement was recorded and named ‘CBP’ (still at normothermia). During the cooling phase, further dissection of the aorta and its branches was performed to ensure a full mobilisation of the structures. Cooling target temperature was either 20°C or 25°C. Cooling times were at least 20 minutes and no longer than 23 minutes. CBFv measurements were recorded when body temperature reached 30°C, 25°C and the lowest temperature. Once the patient was cooled to 28°C, the perfusion strategy to manage blood gases (pO_2_ and pCO_2_) was switched from alpha-stat to pH-stat, which is a temperature corrected method. pH-stat management was only used when the patient was cooled at 28°C or lower. At this time pCO_2_ was also temperature corrected.

Only after the target core temperature was reached, the systemic cannula was removed in patients with two arterial cannulas. The aortic cross-clamp was applied, and myocardial arrest/protection was achieved with infusion of cold blood cardioplegia (ratio 1:4 blood to St. Thomas II solution) in the aortic root. Cardioplegia infusion was repeated at 30 minute intervals. The head and neck vessels (innominate artery, left common carotid and left subclavian arteries) were already fully dissected and ready to be snared when the ascending aorta cannula was advanced into the innominate artery to initiate selective antegrade cerebral perfusion (ACP). At this point, a measurement was taken labelled ‘ACP’. The aortic cross-clamp in the descending aorta was then positioned and CPB flow rates were manipulated manually to keep cerebral oxygenation measurements (measured using NIRS) within the ACP target, which was to maintain NIRS within ∼10% of when the patient was placed onto full flow bypass. Five patients underwent complete hypothermic circulatory arrest (mean time 22 ± 37 min). The aorta was transected at the isthmus above and below the duct insertion and ductal tissue completely removed; the arch was opened in the inner curvature to the mid ascending aorta. End-to-end posterior anastomosis was performed between the descending aorta and distal arch. A pulmonary homograft was used for the reconstruction of the aortic arch in all patients. In two patients who underwent the Norwood operation, the pulmonary trunk, previously detached from the pulmonary bifurcation, was anastomosed to the previously positioned pulmonary homograft in the inner curvature of the reconstructed aortic arch. Once accomplished, the arch was deaired by releasing the distal clamp. The clamps from the head and neck vessels were removed. The tip of the aortic cannula was moved back into the aortic arch to perfuse the whole body and heart with blood. Once the suture lines were checked gradual rewarming was induced. If further procedures were needed such as a ventricular septal defect closure or atrial septal defect closure, further doses of cardioplegia were used, and intracardiac repair was accomplished accordingly. In such cases, longer CPB and cross-clamp time was also needed. A measurement was recorded following whole body reperfusion labelled as ‘whole body reperfusion’. Patients were then rewarmed to normothermia. Measurements were recorded at temperatures 25°C, 30°C and 36°C. Once a temperature of 28°C was achieved, the stat management was switched from pH-stats to alpha-stats. When normothermia was reached, the patient was weaned off CPB. A further measurement was recorded and labelled ‘off CBF’ when haemodynamics were stable. All patients recovered in PICU, and the following post-operative day a measurement was recorded labelled as ‘PICU’.

### Statistical analysis

The data were explored for normality using quantile–quantile plots, the data were normally distributed. SPSS (SPSS Version 26, IMB Statistics, USA) was used to perform linear mixed models to account for missing data points. Analysis were exploratory in nature with all variables compared during the entire procedure, which included the time points baseline, CBP, during cooling at 30°C, 25°C, the lowest temperature, during cerebral perfusion, whole body perfusion, during rewarming at 25°C, 30°C, 36°C, once off CPB and after surgery on PICU (12 time points) and between *a priori* target temperatures of 20°C and 25°C (2 temperatures). Follow-up post hoc comparisons to explore main effect of time were defined *a priori*. Each time point was compared to both baseline and the initiation of CBP (time points used to define cerebral perfusion during the procedure). To examine the correlation between CBFv and NIRS repeated measures correlations (rmcorr) were employed using R statistical package (RStudio: Integrated Development Environment for R, USA). The repeated measures variable was time that included 12 levels. Main effects, time–temperature interactions and correlations demonstrating a *P* value of <0.05 were considered statistically significant.

## RESULTS

A total of 24 patients with a mean age of 19 (±7) days, weighed 3.63 (±0.66) kg and height 51.8 (±3.4) cm were recruited (Table 1). All patients eligible were recruited with no patients withdrawing from the study. Patients had missing TCD data (*n* = 6) and missing NIRS values (*n* = 7) at a single time point. All patients had a follow-up measurement taken on PICU the following day and there were no dropouts. There were no reports of neurological events during cardiac surgery or recovery on PICU.

**Figure 1:**
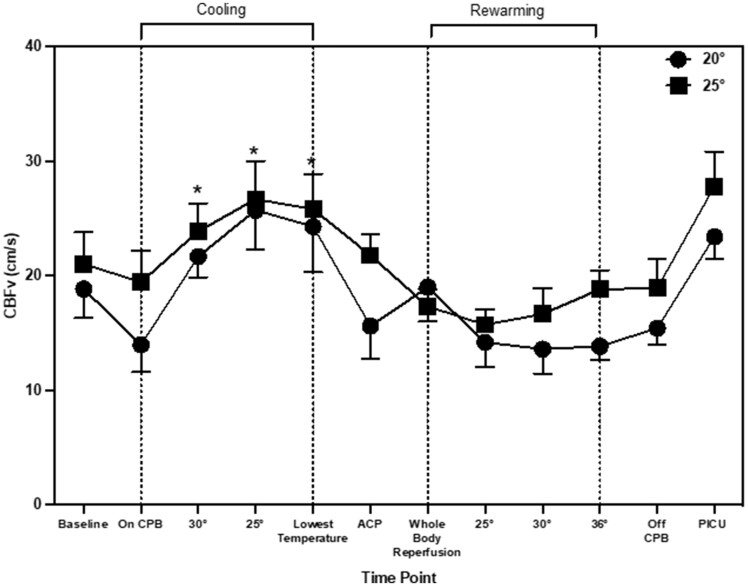
Mean and standard deviation of cerebral blood flow velocity throughout aortic arch repair. * indicates significant change from ‘baseline’ time point.

**Figure 2:**
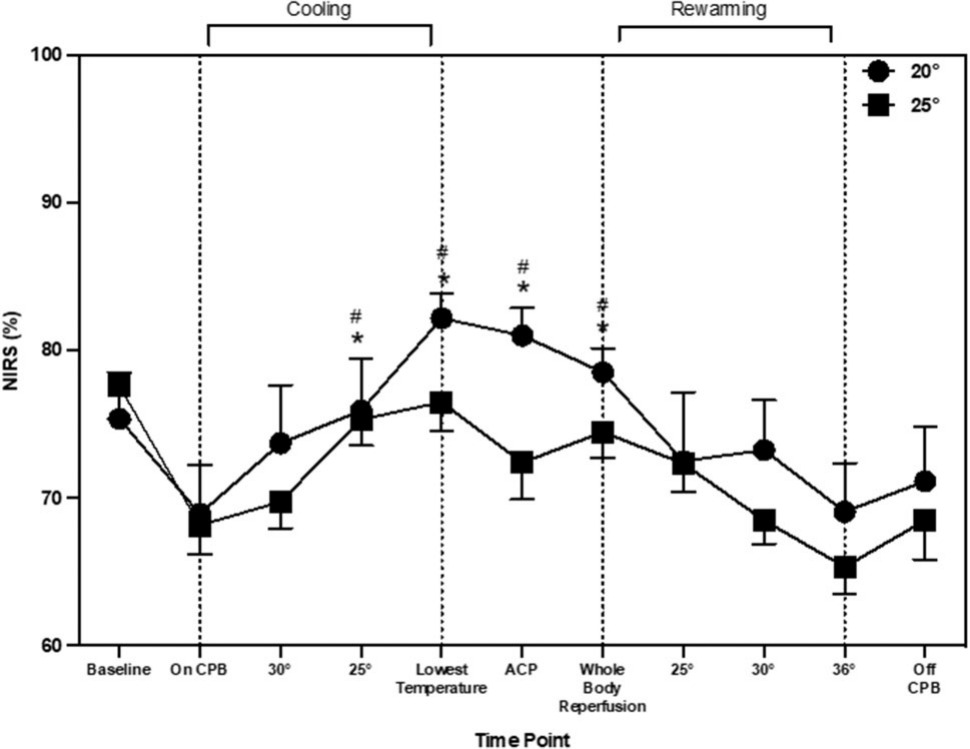
Near infrared spectroscopy throughout aortic arch repair, represented as mean and standard deviation. * shows significant difference from ‘baseline’. # indicates significant difference from ‘on CPB’ time point.

CBFv changed during the entire arch repair procedure (main effect of time: *P* = 0.001). During cooling, CBFv increased by 6.6 cm/s (2.7, 12.8), 10.0 cm/s (5.97, 17.7) and 9.5 cm/s (5.85, 16.6), at 30°, 25°C and lowest temperature, respectively when compared to during CPB (*P* = 0.019). Once recovering in PICU, CBFv increased from the baseline by 6.2 cm/s (0.21, 13.4; *P* = 0.045) and by 7.1 cm/s (1.2, 12.1; *P* = 0.005) from CPB (Fig. 1). CBFv changes were similar between patients cooled to the *a priori* agreed 20°C and 25°C (main effect of temperature: *P* = 0.22; time–temperature interaction; *P* = 0.86). NIRS values changed during aortic arch repair procedure (main effect of time: *P* = 0.028). NIRS increased during cooling from CPB by 10% [3, 11], 7% [7, 13] at 25°C and the lowest temperature (Fig. 2). There was also an increase from CPB at ACP by 8% [4, 12] and whole body reperfusion by 8% [4, 12]. NIRS values were similar between patients cooled to the *a priori* 20°C and 25°C (main effect of temperature *P* = 0.19; time–temperature interaction; *P* = 0.59). Rmcorr identified statistically significant but weak positive correlations between CBFv and NIRS (*r* = 0.25, *P* = 0.001). The CPB flow rates were maintained within the start of CPB except for during ACP where they were reduced by 0.26 l/min^−1^ (0.29, 0.41). The main effect of time (*P* = 0.001), temperature (*P* = 0.47) and time–temperature (*P* = 0.49).

**Figure 3:**
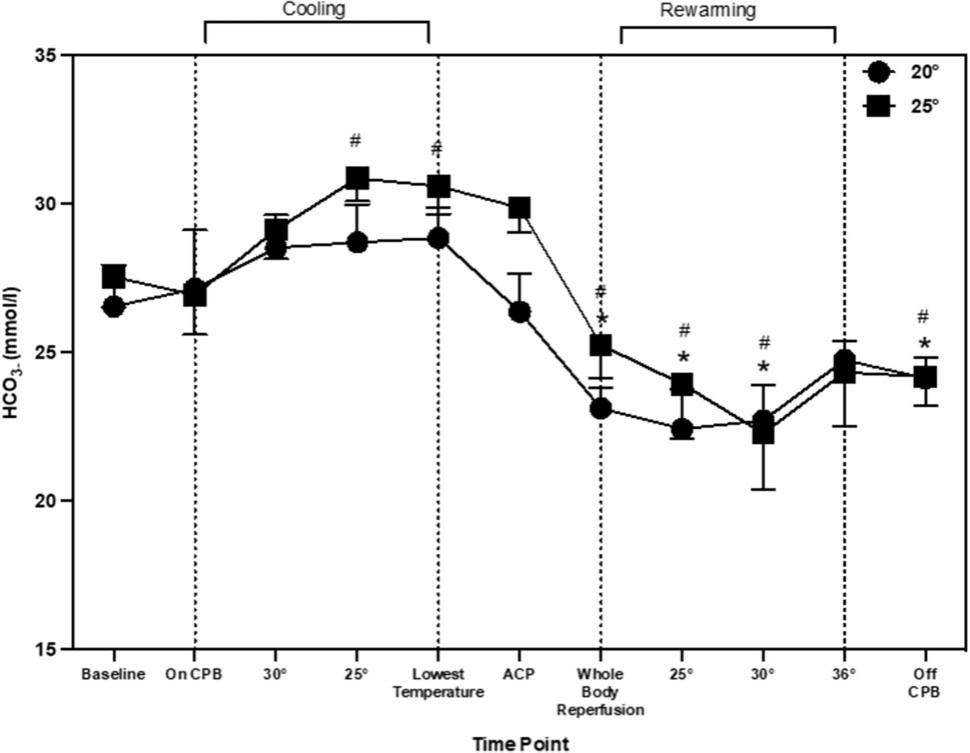
Bicarbonate throughout aortic arch repair represented as means and standard deviation. * shows significant difference from ‘baseline’. # indicates significant difference from ‘on CPB’ time point.

Serum lactate changed during aortic arch repair procedure (main effect of time: *P* = 0.001). When compared to baseline, lactate increased by 2.1 mmol/l (0.93, 2.85) and remained significantly higher throughout the procedure. Compared to CPB, lactate increased by 1.4 mmol/l (0.08, 2.01; *P* = 0.010) at 30°C. The 25°C temperature group elicited the highest lactate levels, but there was no interaction between time and temperature (*P* = 0.60). HCO_3–_ changed during aortic arch repair procedure (main effect of time; *P* = 0.001). When compared to baseline, HCO_3–_ increased during cooling at 25°C and the lowest temperature by 3.2 mmol/l (0.03, 4.72) and 3.1 mmol/l (0.50, 4.70; *P* = 0.002), respectively; then decreased at whole body reperfusion, rewarming at 25°C, 30°C and when off CPB, by 2.0 mmol/l (0.60, 5.29), 3.5 mmol/l (0.78, 7.45), 4.3 (1.27, 8.11) and 2.2 mmol/l (0.92, 5.21), respectively (Fig. 3). When compared to CPB, HCO_3–_ decreased during whole body reperfusion, 25°C, 30°C and off CPB by 2.1 mmol/l (0.07, 5.78), 3.6 mmol/l (0.29, 7.76), 4.4 mmol/l (0.79, 8.42) and 3.2 mmol/l (0.20, 5.75), respectively. HCO_3–_ changes were similar between patients cooled to 20°C and 25°C (main effect of temp; *P* = 0.094, time–temperature; *P* = 0.92).

pO_2_ changed throughout aortic arch repair (main effect of time: *P* = 0.001). Compared to baseline pO_2_ increased by 11.6 kPa (3.10, 15.9) on CPB (*P* = 0.005) and by 8.7 kPa (1.47, 14.6) at 30°C. pO_2_ remained lower from 25°C during cooling until off CPB. pO_2_ changes were similar between patients cooled to the *a priori* 20°C and 25°C (main effect of temperature *P* = 0.37; time–temperature interaction: *P* = 0.16). pCO_2_ changed throughout aortic arch repair (main effect of time; *P* = 0.009). Compared to baseline pCO_2_ decreased (*P* = 0.001) by 2.3 kPa (0.93, 3.43) at CPB, by 1.8 kPa (0.12, 2.64) at 30°C, by 1.9 kPa (0.12, 2.64) at 25°C, by 1.7 kPa (0.33, 2.75) at the lowest temperature of cooling, 1.8 kPa (0.40, 2.85) at ACP, 1.6 kPa (0.61, 2.55) at 30°C and by 1.8 kPa (0.43, 2.89) at 36°C. Compared to baseline pCO_2_ increased by 0.5 kPa (0.21, 1.36) at 30°C, by 0.4 kPa (0.10, 0.97) at lowest temperature, by 0.5 kPa (0.17, 1.11) at ACP, by 1.2 kPa (0.43, 1.07) at whole body reperfusion, by 0.9 kPa (0.63, 1.97) at 25°C, by 0.7 kPa (0.19, 1.55) at 30°C and by 1.0 kPa (0.21, 1.89) off CPB. pCO_2_ changes were similar between patients cooled to the *apriori* 20°C and 25°C (main effect of temperature *P* = 0.47; time–temperature interaction; *P* = 0.22).

Htc changed during aortic arch repair procedure (main effect of time: *P* = 0.001). Htc decreased by 5.5% (0.72, 8.92) when on CPB compared to baseline and remained significantly lower until off CPB. When compared to CPB there was an increase during whole body reperfusion 3.7% (0.72, 6.59), 30°C by 3.6% (0.60, 5.92) and when placed off CPB by 5.0% (0.18, 10.2). There was no main effect of temperature (*P* = 0.17) or time–temperature interaction (*P* = 0.96). Hb changed during aortic arch repair procedure (main effect of time: *P* = 0.003). Hb decreased by 1.4 g/dl (0.06, 2.40) when on CPB and remained lower until off CPB. When compared to CPB, Hb decreased by 1.5 g/dl (0.02, 2.20) at ACP, 1.7 g/dl at whole body reperfusion (0.48, 2.54) and 1.6 g/dl at 25°C rewarming. With a main effect of temperature being higher in the group cooled to 20°C (*P* = 0.011) and main effect of time–temperature (*P* = 0.74).

## DISCUSSION

The aims of this study were threefold: (i) to continuously monitor CBFv throughout aortic arch repair surgery, (ii) investigate the relationship between TCD and NIRS, (iii) compare CBFv in patients cooled to 20°C and 25°C as part of the neuroprotection regime. The current data indicated (i) during the cooling process there was an increase in CBFv and NIRS, although NIRS appears to have a delayed response following increase in CBFv and (ii) that cooling to either 20°C or 25°C did not alter the CBFv response during aortic arch repair surgery. Taken together, the results imply cerebral perfusion is maintained despite deeper cooling in the more complex and longer surgical time periods. Nevertheless, during the cooling process there are physiologically important changes in CBF, which NIRS alone may not be able to detect.

A novel observation from the current data shows that NIRS values increased during the cooling period. A previous study has also reported an increase in cerebral oxygenation from 63 ± 11 to 88 ± 7% after 15 min of cooling during cardiac surgery [14]. In the current study, there was also a significant increase in TCD values during cooling which corresponds with other previous research into paediatric aortic arch repair [9]. It appears that the changes during cooling are not mediated through CPB flow rates as these remained the same throughout the cooling period. There are other possible physiological explanations for this change in perfusion during cooling. First, the type of stat management used may account for these changes. In the current study, once the patients were cooled to 28°C, pH-stat management was used rather than alpha-stats. pH-stat management is a standardised practise used at the centre, the purpose of switching stat management at lower temperatures is to increase tissue oxygenation during DHCA. pH-stat management consists of maintaining pH at ∼7.4 at lower temperatures. To achieve this, CO_2_ was added to the CPB oxygen admixture in the oxygenator (pCO_2_ is kept at ∼40 mmHg) [15]. CO_2_ has been identified as a key regulator of cerebral perfusion, increases in CO_2_ lead to increases in CBF [13]. One previous study measured TCD differences between pH and alpha-stat management during CPB [16]. During pH-stat management, there was a global CBF and MCA velocity increase at 28°C by 45.9 and 51.8%, respectively. Whereas in patients who underwent alpha-stat management throughout cooling, CBF and MCA velocity had a decrease by 26.4 and 22.4%, respectively at 28°C. In the current study, CBFv had already increased at 30°C, therefore stat management was not the only contributing factor. Another possible factor for the increase in CBFv during cooling could be changes in HCO_3–_. Figs 1 and 3 that changes in CBFv and HCO_3–_ follow a similar pattern throughout aortic arch repair. The impact of HCO_3–_ on CBFv has recently been highlighted as an important factor in cerebral autoregulation [17]. Changes occur through the bicarbonate buffering system, with an increase in HCO_3–_ there is an increase in pCO_2_ and hydrogen ions. This activates the gated calcium channels, hyperpolarising the endothelial cells leading to vascular vasodilation in the arterioles and precapillary sphincter [18]. This has been suggested to be more predominant in sedated populations [19]. In non-sedated individuals, with an increase in pCO_2_, usually there is a response to increase breathing to reduce the amount of pCO_2_ in the blood. This is unable to happen in sedated individuals with muscle relaxants, such as during paediatric cardiac surgery. This may provide insight into the pattern of change seen in CBFv during paediatric aortic arch repair.

Cerebral perfusion measurements obtained during ACP demonstrated a decrease in CBFv. Interestingly, NIRS values remained elevated. The current data suggests that NIRS and CBFv does not follow the same pattern during ACP. One potential physiological explanation for the elevated oxygenation despite reductions in CBFv could be the reduced brain metabolism as a result of cooling [20]. This may suggest that despite a reduction in CBFv, cerebral oxygenation remains elevated. Another novel aspect of this study was CBFv measurements taken during rewarming and subsequently in PICU. CBFv values obtained in PICU were higher when compared to baseline and CBP. Post-surgery CBFv values were comparable to normative CBFv in healthy neonates [11].

We also aimed to examine the overall relationship between TCD and NIRS at each time point during surgery. Previous studies have suggested a moderate and statistically significant correlation (*r* = 0.55, *P* < 0.0001) between TCD and NIRS during CPB in adult cardiac surgery [21]. However, a number of concerns have been raised about solely using NIRS as a clinical tool during sedation such as the inability to measure hyperperfusion [7]. In the current study, rmcorr were employed to examine the relationship at each time point during surgery. Rmcorr was employed as it averages repeated measures for each individual, therefore the assumption of independence is not violated using this method [22]. The current data suggested a statistically significant but weak correlation. Physiologically, even small changes in CBFv could have an impact on cerebral perfusion, which could potentially be underestimated by NIRS. This discrepancy was highlighted at the time point named ‘ACP’, where CBFv values decreased and NIRS remained elevated.

During this time, the discrepancy may not necessarily be detrimental. Similar findings were documented during neonatal cardiac surgery, where despite a reduction in CBFv at ACP, levels of oxygenation remained elevated [23] with the conclusion that the requirement of oxygenated blood decreases during DHCA. However, this finding has highlighted a discrepancy between CBFv and NIRS, which may have clinical importance at time periods outside of DHCA. Taken together, the additional measurement of TCD alongside NIRS might be beneficial to indicate global brain perfusion in this patient group, as opposed to NIRS alone that can only give regional measurements of the frontal lobes. TCD can also indicate hyperperfusion which NIRS cannot. This would give further information about perfusion in the main cerebral arteries and reduce the likelihood of perfusion injuries during CPB.

The final novel aspect of the current study was examining the differences between two temperature groups. Currently in clinical care, cooling patients to specific target temperature is based on surgeon or centre preference. The rationale for cooling temperatures of 18°C or 20°C during aortic arch repair is to maximise neurological protection during complex and longer operations. Data from the current study suggested there was no significant difference in CBFv or NIRS between patients cooled to 20°C or 25°C. Using a higher temperature such as 25°C might prevent the systemic drawback of perfusing the body at lower temperatures. Moreover, it could be beneficial in reducing the cooling and rewarming times, hence allowing for a shorter CPB time. However, this would need to be explored further in subsequent studies to impact clinical practise.

### Methodological considerations and limitations

A limitation to the study was the relatively small sample size as this was a single-centred study, future research should consider a larger study. A limitation of TCD is the inability to measure vessel diameter, therefore absolute flow cannot be quantified. However, during a rested state vessel diameter is expected to be maintained, meaning TCD can give an accurate indication of flow. The anaesthetic technique was formulated to have minimal effect on vessel diameter and CBF. Previous research has suggested that the use of fentanyl and inhaled sevoflurane does not affect CBF [24], and midazolam has minimal effect on CO_2_ reactivity, CBF and blood oxygenation [25]. Future studies should consider measuring vessel diameter to ensure diameter remains constant during cardiac surgery. Another limitation of TCD is that it is operator dependent. To minimise, all CBFv measurements were taken by one trained individual. The final limitation is the inclusion of two patients with HLHS. It was acknowledged that patients with HLHS have differing cardiac physiology. However, the inclusion criteria stated any patient who was undergoing arch repair using a hyperthermic circulation. HLHS patients met this criterion. It was also important to the main aim of the study to capture these patients as those cooled to lower temperatures are often undergoing more complex procedures such as the Norwood (which includes Damus Kaye Stansel anastomosis needing longer CPB and ACP times). It is important to highlight, these patients had similar clinical interventions with prostaglandin being used to keep the PDA open and ages were comparable to other patients enrolled. Once they were on CPB, flow rates were maintained as per general protocol. During the Norwood operation with Sano modification, it was anticipated that CBFv levels would be comparable across all patients, irrespective of physiology. As this was a feasibility study, it was believed it was important to capture these patients so the comparison between the two temperatures could be included in the analysis, which is a novel aspect of the study.

## CONCLUSION

The main findings suggested that during the cooling process there was an increase in both CBFv and NIRS, although there appears to be a delayed response in NIRS. Although this delay did not appear to be detrimental during cooling due to the reductions in brain metabolism. But it has highlighted that NIRS alone may not be truly reflective of cerebral perfusion, despite its current heavy reliance during cardiac surgery. This corresponds with the finding of an overall weak relationship between TCD and NIRS. Another key finding was that cooling to 20°C or 25°C did not alter the CBFv response during neonatal aortic arch repair surgery. Overall, these findings could provide clinicians with information on how to optimise long-term cerebrovascular health in this population.

## Data Availability

The data underlying this article will be shared on reasonable request to the corresponding author.

## Notes

Presented at the ECHSA Annual Meeting (remote), 2021.

## ABBREVIATIONS

ACP: Antegrade cerebral perfusion
CBF: Cerebral blood flow
CBFv: Cerebral blood flow velocity
CPB: Cardiopulmonary bypass
DHCA: Deep hypothermic circulatory arrest
Hb: Haemoglobin
HCO_3_: Bicarbonate
Htc: Haematocrit
MCAv: Middle cerebral artery velocity
MCA: Middle cerebral artery
NIRS: Near-infrared spectroscopy
PICU: Paediatric intensive care unit
pCO_2_: Partial pressure of carbon dioxide
pO_2_: Partial pressure of oxygen
PDA: Patent ductus arteriosus
rmcorr: Repeated measures correlations
TCD: Transcranial doppler
